# Identification and functional analysis of long non‐coding RNAs in the synovial membrane of osteoarthritis patients

**DOI:** 10.1002/cbf.3491

**Published:** 2020-01-20

**Authors:** Xiaolong Shui, Qipeng Xie, Shaomin Chen, Chengwei Zhou, Jianzhong Kong, Yi Wang

**Affiliations:** ^1^ Department of Orthopedics The Second Affiliated Hospital and Yuying Children's Hospital of Wenzhou Medical University Wenzhou China; ^2^ Department of Laboratory Medicine The Second Affiliated Hospital and Yuying Children's Hospital of Wenzhou Medical University Wenzhou China; ^3^ Department of Rehabilitation The Second Affiliated Hospital and Yuying Children's Hospital of Wenzhou Medical University Wenzhou China

**Keywords:** hub gene, lncRNA, NONHSAG034351, osteoarthritis, WGCNA

## Abstract

Osteoarthritis (OA), the most common chronic joint disease in the elderly, has become a significant economic burden for families and societies worldwide. Although treatments are continually improving, current drugs only target joint pain, with no effective therapies modifying OA progression. Long noncoding RNAs (lncRNAs), which have received increasing attention in recent years, are abnormally expressed in OA cartilage. In the present study, weighted coexpression network analysis (WGCNA) was applied to identify modules related to certain OA clinical traits. In total, 4404 coding genes and 161 lncRNAs were differentially expressed based on two OA expression profile data sets and normal control samples. Subsequently, 11 independent modules were acquired, and the green module, with a total of 49 hub genes, was identified as the most relevant to OA. These hub genes were validated using the GSE12021 data set. There was only one lncRNA among the hub genes, namely, NONHSAG034351. Thus, we further explored the function of NONHSAG034351‐related genes in the network. Gene Ontology (GO) enrichment and Kyoto Encyclopedia of Genes and Genomes (KEGG) pathway analysis showed that NONHSAG034351‐associated genes are involved in the response to lipopolysaccharide, angiogenesis, tumour necrosis factor (TNF) signalling, and mitogen‐activated protein kinase (MAPK) signalling pathways. In conclusion, we identified modules through WGCNA related to OA clinical traits. NONHSAG034351, the only hub‐lncRNA, was downregulated in OA synovial tissue and might play a significant role in the pathological progression of this disease. Our findings have important clinical implications and could provide novel biomarkers that indicate the molecular mechanisms of OA and act as potential therapeutic targets.

**Significance of this study:**

Long noncoding RNAs (lncRNAs) have been reported to be abnormally expressed in osteoarthritis (OA), which is the most common chronic joint disease among the elderly. In the present study, we report the expression profiles of lncRNAs in OA and the identification of modules through WGCNA related to OA clinical traits. NONHSAG034351, the only hub‐lncRNA identified to be downregulated in the synovial tissue of OA patients, might play a significant role in the pathological progression of OA. Furthermore, our findings provide novel biomarkers associated with the molecular mechanisms underlying OA pathogenesis, thus implying potential therapeutic targets with important clinical implications.

## 1 INTRODUCTION

Osteoarthritis (OA) is the most common chronic joint disease in older adults and is characterized by the degeneration of articular cartilage, subchondral bone sclerosis, osteophyte formation, and synovial membrane inflammation, which causes joint pain, joint dysfunction, and disabilities.^2^, ^7^, ^30^ Globally, 250 million people are estimated to have OA of the knee, and the incidence is increasing.^42^, ^43^ Moreover, because of medical costs and lost wages, OA has become a tremendous economic burden for families and societies worldwide.^40^ Although treatment options are continually improving, current therapies cannot effectively modify OA progression.^33^ The pathogenic mechanism of OA is complicated and multifactorial, being related to genetic, biological, and immunologic factors.^20^ Recent studies have shown an association between epigenetics and OA, in which noncoding RNAs (ncRNAs) were found by microarray analysis to participate in the processes of cartilage and bone development.^6^, ^37^

Long ncRNAs (lncRNAs), comprising 200‐nucleotide long ncRNAs, are mRNA‐like molecules and widely transcribed in mammalian genomes.^10^ In the past decade, lncRNAs have garnered increasing attention and have been confirmed to participate in different biological processes, serving as signals, decoys, guides, and scaffolds.^45^ Studies have also shown that lncRNAs are abnormally expressed in various human diseases and affect their development.^32^ For example, lncRNA‐HOTAIR and lncRNA‐CIR are aberrantly expressed in OA cartilage, suggesting that they might have significant effects on the diagnosis and prognosis of OA and could be used as personalized therapeutic biomarkers to indicate OA progression.^3^, ^14^, ^26^ However, current studies have mainly focused on a single genetic event or a single cohort. The lncRNA expression profiles and related mRNA expression patterns, as well as their potential biological functions in OA, remain unclear.

Recently, novel bioinformatics and computational technologies have been widely applied to human diseases.^50^ Weighted coexpression network analysis (WGCNA) is a systematic biological approach to identify the relationship between genes based on microarray or RNAseq data.^19^, ^21^ It explores the complex relationships between gene expression and clinical traits by transforming the gene expression data into a coexpression module.^44^ WGCNA has been widely used to investigate different biological processes and has identified several potential biomarkers and therapeutic targets.^35^, ^56^ In this study, we integrated two datasets, GSE55235 and GSE55457, from the Gene Expression Omnibus (GEO) database and performed WGCNA to identify useful modules and potential lncRNAs related to OA. Further, Gene Ontology (GO) enrichment and Kyoto Encyclopedia of Genes and Genomes (KEGG) pathway analysis of the coexpressed genes in the most significant module was performed to explore the biological functions of the identified lncRNA. Our study aimed to construct a network between coexpressed genes based on profile datasets from multiple cohorts, which might promote the identification of important modules and lncRNAs related to OA to provide potential therapeutic biomarkers for its clinical treatment.

## 2 MATERIALS AND METHODS

### 2.1 Data collection

The raw data sets used in this study were downloaded from the GEO database (http://www.ncbi.nlm.nih.gov/geo/). Data sets GSE55235, GSE55457, and GSE12021 were obtained using the Affymetrix Human Genome U133 A Array (HG‐U133A) platform, using RNA extracted from the synovial tissue of OA patients and normal controls. Synovial membrane samples were obtained either from postmortem joints and traumatic joint injury patients (in the case of normal control samples) or from OA patients upon joint replacement/synovectomy. Tissues were homogenized before total RNA isolation. Then, gene expression was analysed using HG‐U133A RNA microarrays. The original study was approved by the respective ethics committees (Jena University Hospital: Ethics Committee of the Friedrich Schiller University Jena at the Medical Faculty; Charité‐Universitätsmedizin Berlin: Charité Ethics Committee; and University of Leipzig: Ethics Committee at the Medical Faculty of the University of Leipzig), and informed patient consent was obtained.^49^ All three data sets were from the same multicenter study and were generated by different laboratories. Because the GSE55584 data set does not contain any control samples, only data sets GSE55235 and GSE55457 were selected for further analysis. The data sets GSE55235 (10 OA and 10 normal control synovial tissues) and GSE55457 (10 OA and 10 normal control synovial tissues) were used to construct the gene coexpression network and identify genes related to OA. These identified hub genes were validated using the data set GSE12021 (which included 10 OA and 9 normal control synovial tissues), another independent data set from a study by Huber *et al*.^16^

### 2.2 Array probe annotation and differential expression analysis

In this study, a customized reannotated chip‐description‐file (CDF) of HG‐U133A microarrays was generated as previously described.^24^ In brief, the probe sequences of HG‐U133A microarrays provided by Affymetrix were aligned to lncRNA transcript sequences (from NONCODEv5) and to the coding gene transcript sequences (from the RefSeq database^36^) using BLASTn. Criteria for transcripts were as follows: (1) only perfectly matched probe‐transcript pairs were retained; (2) probes were removed by targeting both lncRNA and coding transcripts; (3) all transcript sequences corresponding to the retained probe‐transcript pairs were mapped to the hg19 reference genome and annotated at the gene level; (4) both coding and lncRNA genes detected by ≥4 probes were included. Then, a reannotated CDF package (corresponding to the old CDF package HG‐U133A.cdf provided by Affymetrix) was generated using the “makecdfenv” R package.

Using this new CDF file, a total of 9775 coding and 526 lncRNA genes were annotated. The Robust Multiarray Average (RMA) algorithm in the Affy package was used to correct for background, as well as for log_2_ transformation and quantile normalization of our data sets. The limma Bioconductor R package was employed to determine the differentially expressed genes between OA and normal control synovial tissues by one‐way analysis of variance (ANOVA), and the false discovery rate (FDR) was corrected by the Benjamini‐Hochberg method. Using the differential gene expression data (*P* value <.05), all samples were clustered through the Euclidean distance method, and unaggregated samples were removed from the cluster. Finally, GSM1337307 from GSE55457 was removed. Genes with a fold change >1.5 and a *P* value <.05 were defined as significant differentially‐expressed genes.

### 2.3 Construction of a lncRNA‐mRNA weighted coexpression network

The coexpression network for coding and lncRNA genes was constructed using the “WGCNA” R package.^21^ We first selected genes with a *P* value <.05 for a two‐sample ***t***‐test between OA and control samples. In total, 4404 coding genes and 161 lncRNA genes were selected to construct the coexpression network. Then, we removed the batch effect between GSE55235 and GSE55457 using the “remove Batch Effect” function in the limma R package before constructing the coexpression network. Coexpression network construction was performed using the “blockwiseModules” function in the WGCNA package. Briefly, we first calculated the Pearson's correlation coefficient for all possible gene pairs. A weighted adjacency matrix (WAM) was subsequently constructed using a power function, which resulted in a weighted network. After sensitivity analysis of scale‐free topology, the soft‐thresholding power parameter, β, was set to 12. A topological overlap matrix (TOM) and the corresponding dissimilarity (1‐TOM) were calculated based on the WAM.

### 2.4 Identification of clinically significant modules and hub genes

To identify clinically significant modules associated with OA, two methods were used. First, we calculated the log_10_ transformation of the *P* value based on the linear regression between gene expression and the phenotypic trait, named gene significance (GS). The average GS for all genes in a module, named module significance, was also calculated. Then, the module eigengenes (MEs), which were defined as the first principal components for each module, were calculated. The correlations between MEs and clinical OA traits were also calculated to identify the most relevant module to OA. In this study, genes with high GS (> 0.2) and high intramodular connectivity (module membership [MM] > 0.8) in the green module were defined as hub genes. In addition, hub genes were also genes that were significantly differentially expressed.

### 2.5 Functional analysis of lncRNA

The potential functions of the identified lncRNA were predicted based on the functional analysis of coexpressed genes in the green module. GO enrichment and KEGG pathway analyses were performed using the “clusterProfiler” R package.^52^

## 3 RESULTS

### 3.1 Data preprocessing and co‐expression network construction

**Figure 1:**
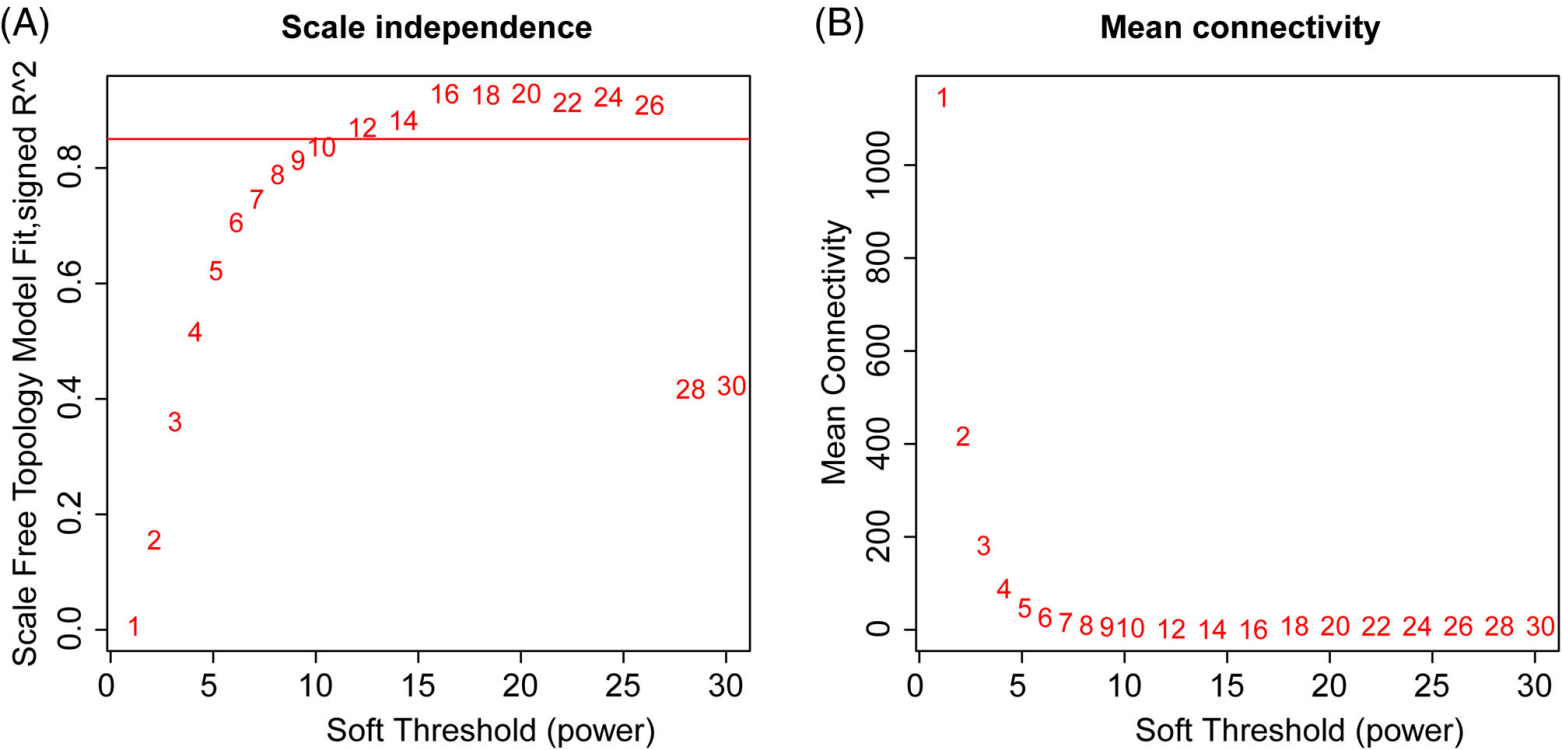
Determination of soft‐thresholding power in the co‐expression network analysis. A, Analysis of the scale‐free fit index for various soft‐thresholding powers (β). B, Analysis of the mean connectivity for various soft‐thresholding powers

**Figure 2:**
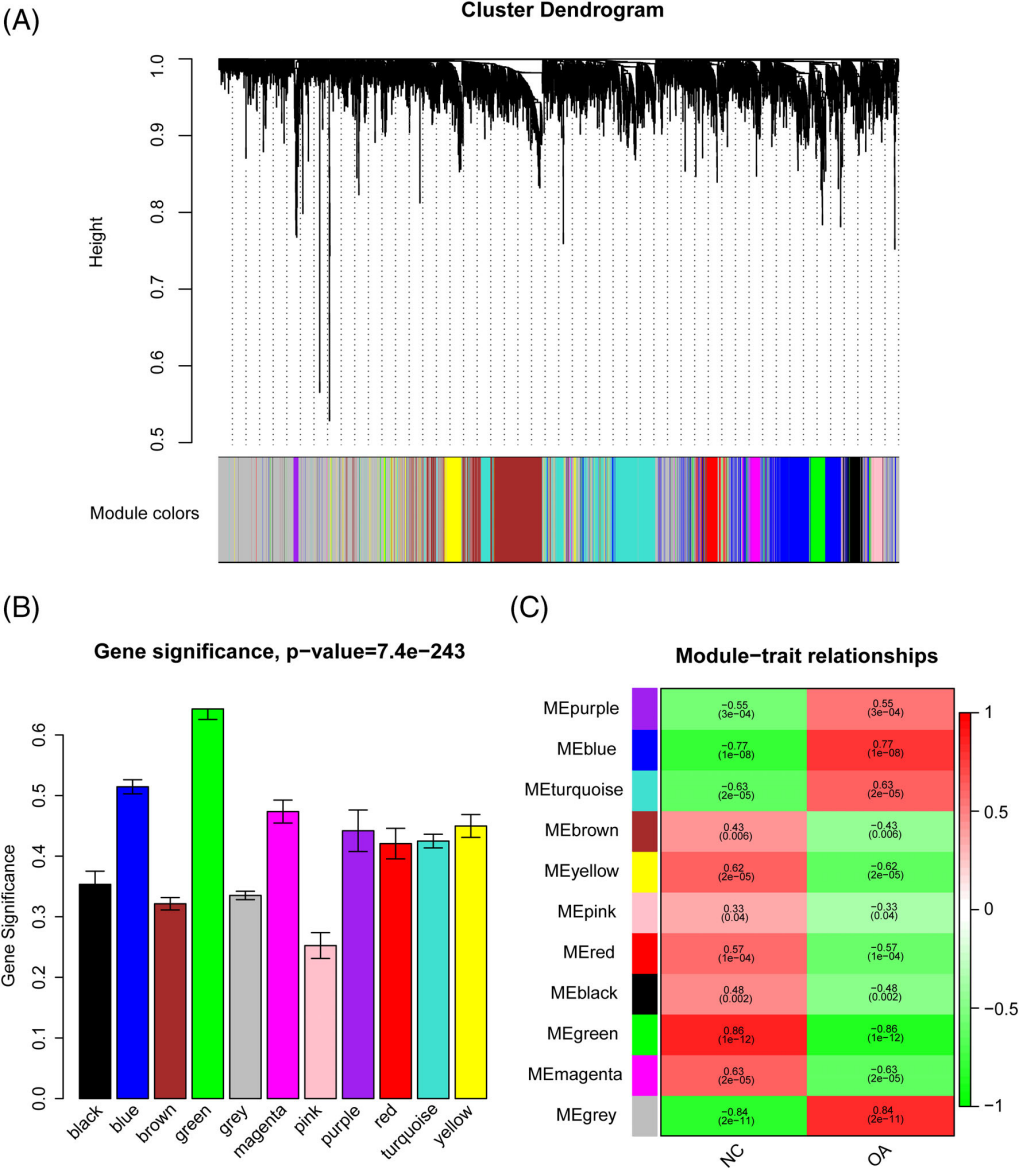
Identification of modules associated with osteoarthritis (OA). A, Dendrogram of 4565 coding/lncRNA genes clustered based on a dissimilarity measure (1‐TOM). B, Bar plot of module significance defined as the mean gene significance (GS) across all genes in the module. The green module was the most promising module associated with OA. C, Heatmap of the correlation between module eigengenes (MEs) and OA

**Figure 3:**
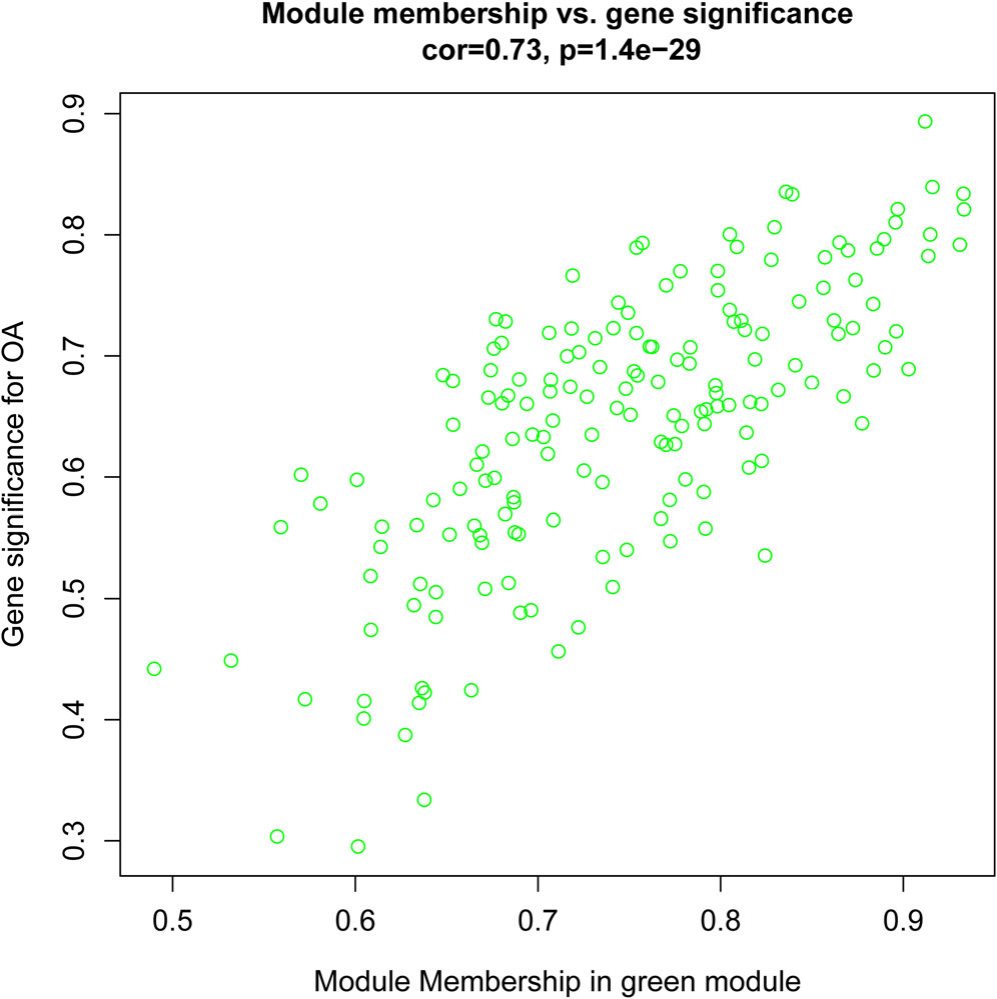
Scatterplot of gene significance (GS) for osteoarthritis (OA) vs module membership (MM) in the green module. There was a highly significant correlation between GS and MM in this module

OA and normal control synovial tissue gene expression profiles of GSE55235, GSE55457, and GSE12021 were obtained from the GEO database. After background correction and quantile normalization of the raw data, the gene expression profiles were calculated using our reannotated CDF file. Next, differentially expressed genes between the OA and normal control synovial tissues were identified using the limma R package. After removal of the batch effect (Supporting Information Figure S1), a total of 4565 differentially expressed genes—4404 coding and 161 lncRNA genes—with a *P* value <.05 in GSE55235 or GSE55457 were used to construct a weighted gene coexpression network using the “WGCNA” package in R. The soft‐thresholding power of β = 12 was selected to ensure a scale‐free network (Figure 1A,B). An average linkage hierarchical clustering tree (dendrogram) was then constructed, and 11 modules with various colours were identified, which can be seen from the coloured‐band underneath the cluster tree (Figure 2A). To explore significant modules associated with OA, we employed two methods (see MATERIALS AND METHODS) that enabled the evaluation of the relationship between each module and OA. We found that the mean GS of the green module was higher than that of any other module (Figure 2B). The ME in the green module also showed a higher correlation with clinical traits than any other module (*R* ^2^ = 0.86, *P* = 1e−12; Figure 2C). In addition, the correlation between GS and MM in the green module was the most significant (Figure 3, Supporting Information Figure S2). Thus, the green module (Supporting Information Table S1) was identified as the most related module to OA.

### 3.2 Identification of hub genes in the synovial tissue of OA patients

**Figure 4:**
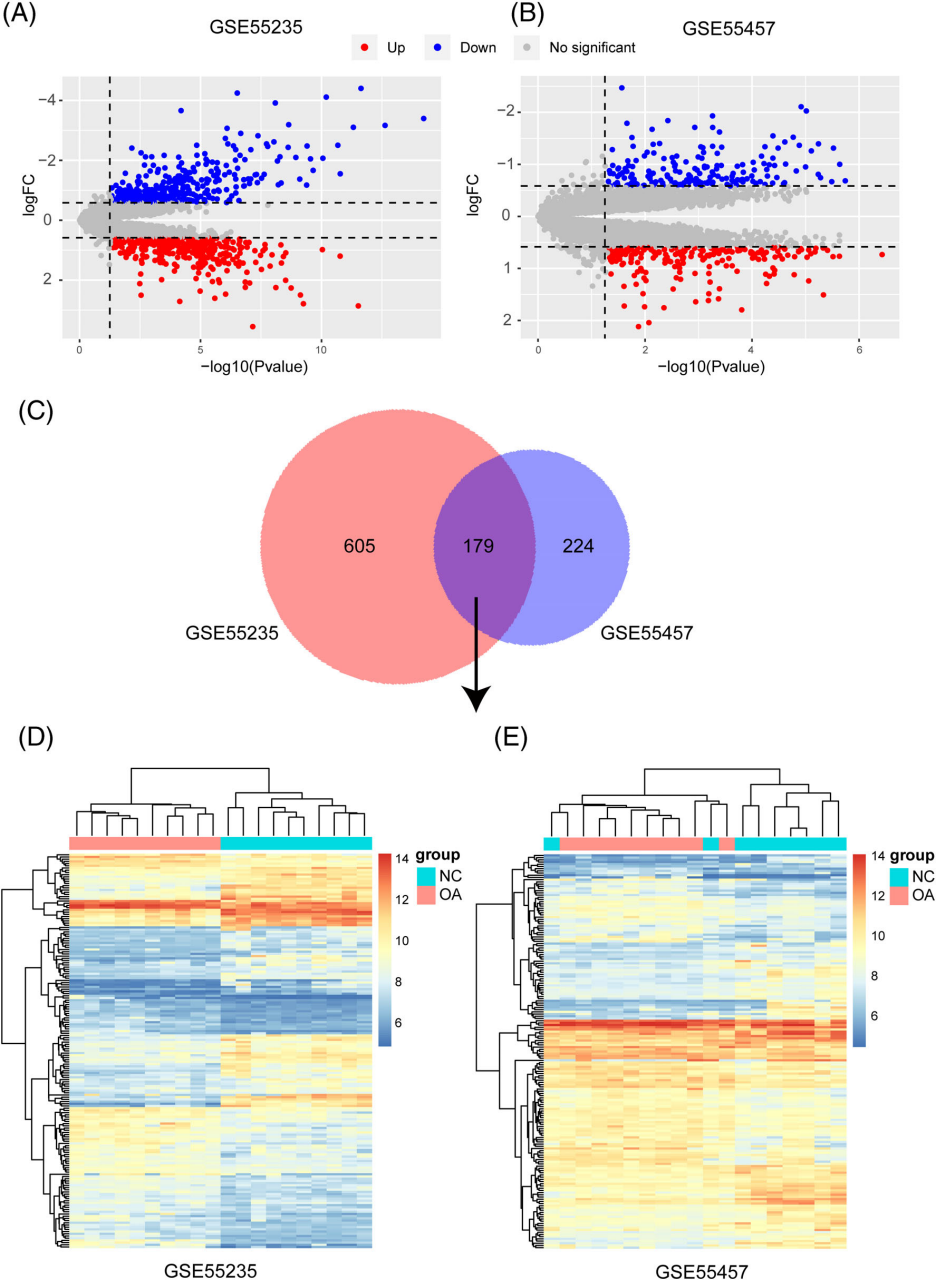
Differentially expressed genes/long noncoding RNAs (lncRNAs) in osteoarthritis (OA) vs normal control samples. (A, B) Volcano plots of fold change and *P*‐values of differentially expressed coding/lncRNA genes in the GSE55235 (A) and GSE55457 (B) data sets. Red points represent upregulated differentially expressed genes. Blue points represent downregulated differentially expressed genes. Grey points indicate coding/lncRNA genes that were not differentially expressed. (C) Venn diagram of differentially expressed coding/lncRNA genes in GSE55235 and GSE55457. (D, E) Heatmaps of differentially expressed coding/lncRNA genes among GSE55235 (D) and GSE55457 (E)

**Figure 5:**
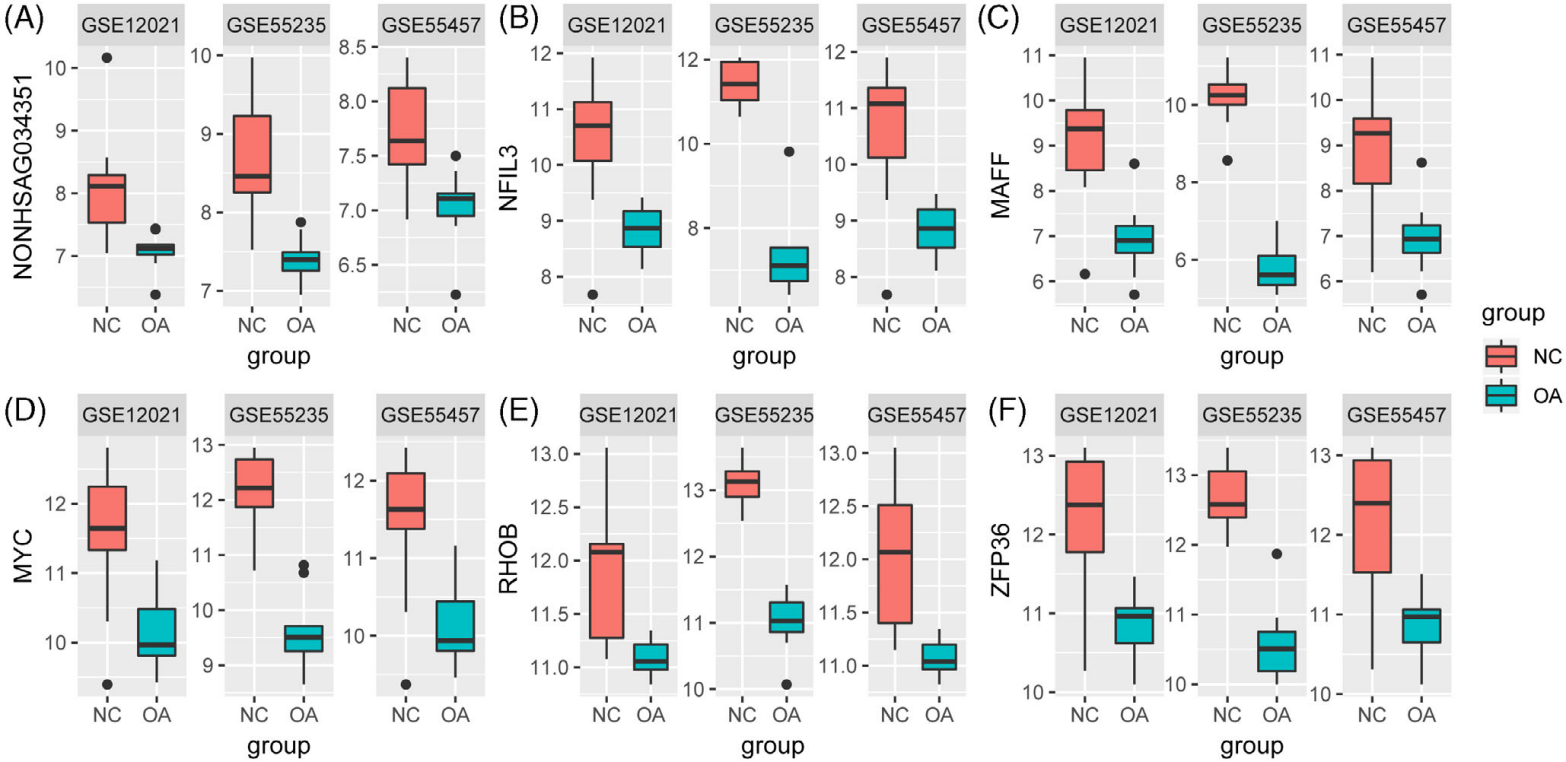
Box plot of the top five hub genes and lncRNA NONHSAG034351 in the GSE55235, GSE55457, and GSE12021 data sets. Validation of the gene expression levels of the top five hub genes and long noncoding RNAs (lncRNA) NONHSAG034351 between normal control (NC) and osteoarthritis (OA) samples in GSE55235, GSE55457, and GSE12021. (A) NONHSAG034351, (B) NFIL3, (C) MAFF, (D) MYC, (E) RHOB, (F) ZFP36

Genes with high MM (weighted correlation >0.8) and GS > 0.2 in the green module were extracted. Then, significant differentially expressed genes in both GSE55235 and GSE55457 data sets were identified. Based on the threshold of >1.5‐fold change and *P* value <.05, we identified 784 differentially expressed genes from the expression profile in the GSE55235 data set, among which 401 were upregulated and 383 were downregulated (Figure 4A, Supporting Information Table S2). In the same way, 403 differentially expressed genes were identified from the GSE55457 data set, among which 184 were downregulated and 219 were upregulated (Figure 4B, Supporting Information Table S3). Further, through integrated bioinformatics analysis, a total of 179 consistently expressed genes were identified from the two profile data sets (Figure 4C, Supporting Information Table S4). In addition, the clusters were significantly separated between the OA group and normal control group, and the expression levels of these genes in the two data sets were significantly different between the two groups (Figure 4D,E). Finally, only the significant differentially expressed genes with MM > 0.8 and GS > 0.2 were identified as hub genes. A total of 49 hub genes were identified in the green module (Table 1). Among these, the top five hub genes and the only lncRNA, NONHSAG034351, were all downregulated in the synovial tissue of OA patients compared with their expression in normal samples in both the GSE55235 and GSE55457 data sets. To further confirm the reliability of the six identified hub genes from the two data sets, we downloaded an additional data set, GSE12021, and found that all six downregulated genes identified in this study were also significantly downregulated in the GSE12021 OA samples (Figure 5).

**Table 1:** Hub genes in the green module

Gene_id	moduleColor	GS.OA	p.GS.OA	MMgreen	p.MMgreen
81575	Green	0.893158	2.09363e−14	0.911050294	8.23792e−16
388	Green	0.838999	2.5565e−11	0.915047824	3.64229e−16
1524	Green	0.835043	3.86639e−11	0.834880089	3.93182e−11
23764	Green	0.833425	4.5647e−11	0.932001968	6.85712e−18
4056	Green	0.833	4.76693e−11	0.838247785	2.76783e−11
150094	Green	0.820722	1.58567e−10	0.89606451	1.2891e−14
4783	Green	0.820546	1.61216e−10	0.932317992	6.30797e−18
687	Green	0.809818	4.28289e−10	0.894840409	1.5839e−14
3725	Green	0.805806	6.07606e−10	0.828544939	7.4537e−11
25864	Green	0.799923	1.00031e−09	0.804128368	7.01624e−10
7538	Green	0.799838	1.00746e−09	0.913832833	4.68719e−16
1052	Green	0.795837	1.40079e−09	0.888564644	4.38187e−14
2355	Green	0.7932	1.73382e−09	0.864161256	1.37877e−12
4609	Green	0.791347	2.01047e−09	0.930055517	1.13672e−17
1316	Green	0.789562	2.31546e−09	0.807983026	5.03082e−10
9314	Green	0.788236	2.56934e−09	0.884689597	7.97217e−14
25976	Green	0.78669	2.89799e−09	0.868801428	7.55167e−13
7128	Green	0.782037	4.13914e−09	0.912775935	5.81963e−16
3727	Green	0.780943	4.49539e−09	0.856188297	3.69035e−12
23099	Green	0.778923	5.22913e−09	0.826799719	8.8495e−11
1051	Green	0.762221	1.72365e−08	0.872888254	4.35949e−13
23645	Green	0.75574	2.6688e−08	0.855339588	4.08386e−12
7832	Green	0.744459	5.53673e−08	0.84201525	1.85109e−11
10560	Green	0.742363	6.31464e−08	0.882579602	1.09455e−13
1847	Green	0.737493	8.52972e−08	0.804059112	7.05783e−10
3491	Green	0.728809	1.435e−07	0.861145991	2.01509e−12
23710	Green	0.728644	1.44898e−07	0.810365214	4.08069e−10
123	Green	0.727783	1.52401e−07	0.806303777	5.82061e−10
9194	Green	0.722502	2.06846e−07	0.871449311	5.30116e−13
4929	Green	0.721011	2.25199e−07	0.812329344	3.42627e−10
5292	Green	0.719863	2.40331e−07	0.895252356	1.47826e−14
3726	Green	0.717751	2.70676e−07	0.821944502	1.41266e−10
2920	Green	0.717718	2.71183e−07	0.863548791	1.49033e−12
9531	Green	0.706626	4.97748e−07	0.88917316	3.98084e−14
NONHSAG034351	Green	0.696661	8.39315e−07	0.817844234	2.07429e−10
9510	Green	0.691795	1.07518e−06	0.839928064	2.31618e−11
10957	Green	0.688592	1.26221e−06	0.901993501	4.57896e−15
694	Green	0.687562	1.3285e−06	0.882845219	1.05209e−13
1105	Green	0.677464	2.1702e−06	0.849029358	8.50626e−12
6617	Green	0.671439	2.88262e−06	0.830610059	6.06843e−11
10221	Green	0.666118	3.68418e−06	0.866403635	1.03362e−12
1880	Green	0.661469	4.547e−06	0.815211317	2.64134e−10
390	Green	0.659839	4.89093e−06	0.821342704	1.4955e−10
1958	Green	0.658972	5.08348e−06	0.803650827	7.3077e−10
5277	Green	0.643862	9.77241e−06	0.876520554	2.63258e−13
10135	Green	0.636214	1.34235e−05	0.813218161	3.16358e−10
1326	Green	0.6129	3.3577e−05	0.821587746	1.46124e−10
6322	Green	0.607355	4.13187e−05	0.814606827	2.79053e−10
10950	Green	0.535093	0.00044854	0.823393998	1.23039e−10

### 3.3 Functional analysis of the lncRNA NONHSAG034351

**Figure 6:**
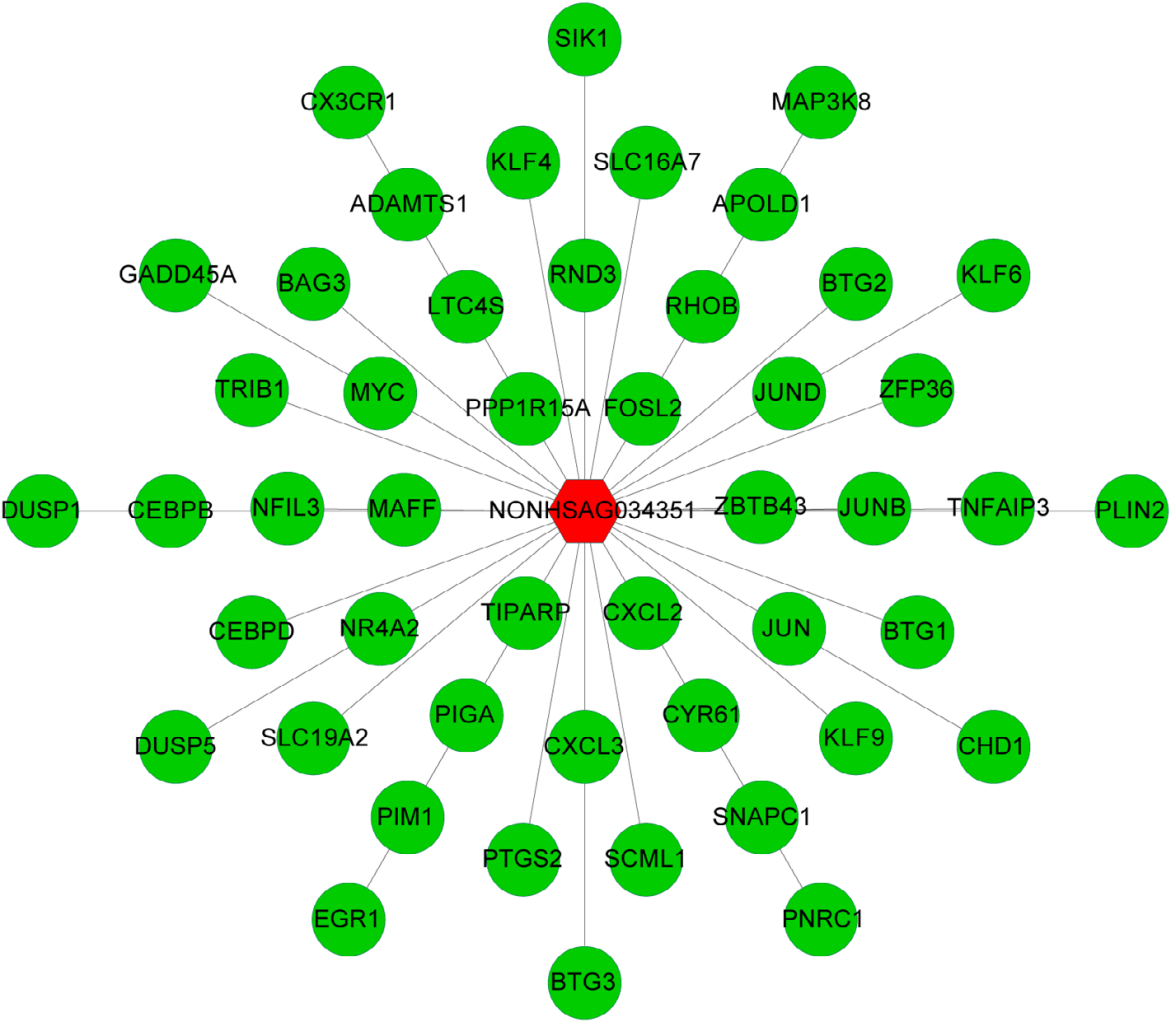
An integrated network of regulatory relationships between long noncoding RNAs (lncRNA) NONHSAG034351 and targeted coding genes in the green module

**Figure 7:**
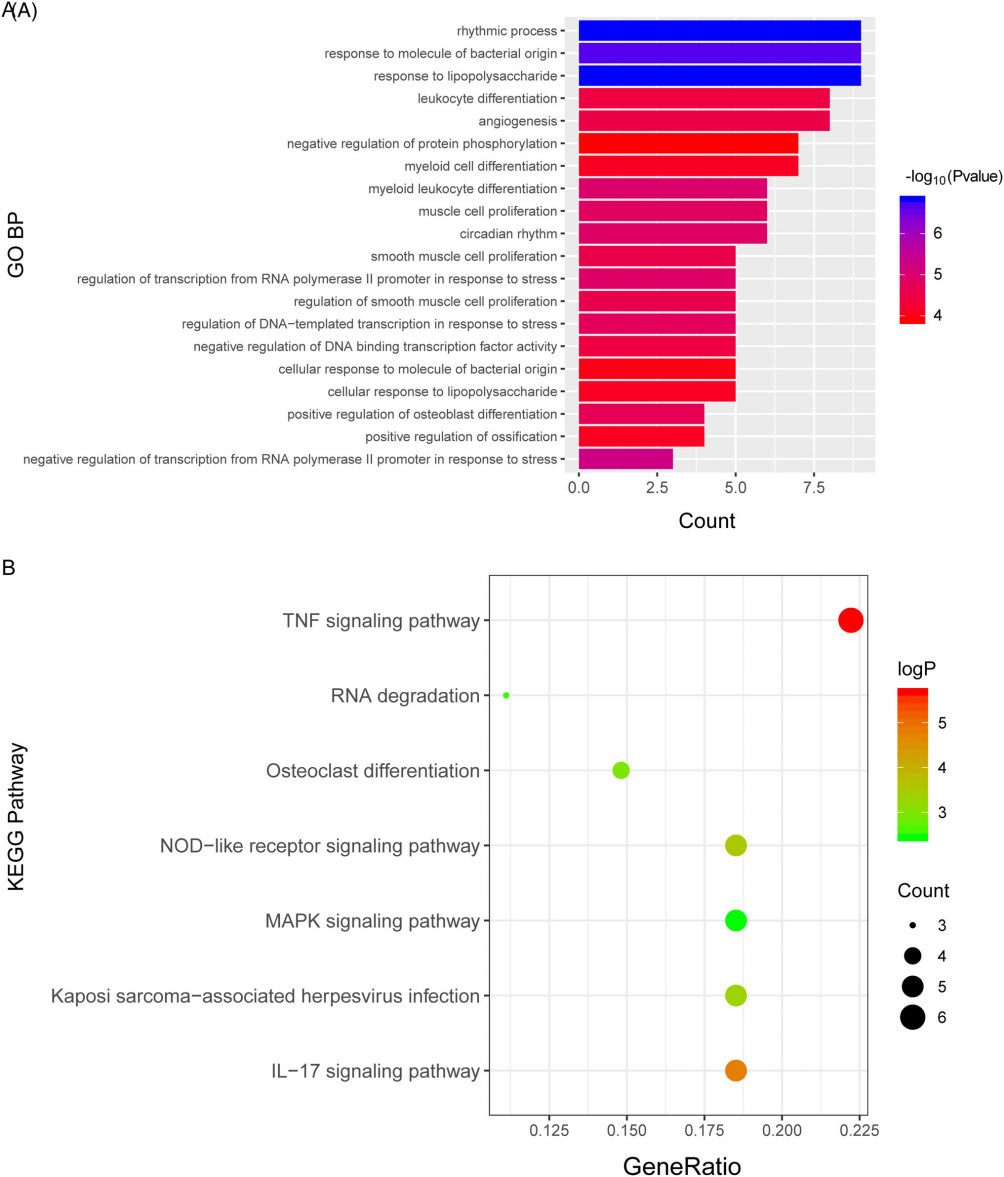
Gene Ontology (GO) enrichment and Kyoto Encyclopedia of Genes and Genomes (KEGG) pathway analysis of co‐expressed hub genes in the green module. (A) Significantly enriched GOBP terms. (B) Significantly enriched KEGG pathways. IL, interleukin; MAPK, mitogen‐activated protein kinase; NOD, nucleotide‐binding oligomerization domain

The only lncRNA, NONHSAG034351, among the 49 hub genes in the green module attracted our attention. There were 48 genes with connectivity to NONHSAG034351, which were regarded as potential targets and coexpressed genes (Figure 6). The biological effects of lncRNAs are mainly mediated by the regulation of their coexpressed or target genes. Therefore, we performed KEGG pathway and GO enrichment analysis on the coexpressed genes of the green module to further explore the biological functions of NONHSAG034351. Among the biology processes, lncRNA NONHSAG034351 mainly participated in the response to lipopolysaccharide (LPS), rhythmic process, response to molecules of bacterial origin, and angiogenesis (Figure 7A, Supporting Information Table S5). The KEGG pathway analysis showed that lncRNA NONHSAG034351 mainly participated in the tumour necrosis factor (TNF), interleukin (IL)‐17, nucleotide‐binding oligomerization domain (NOD)‐like receptor, and mitogen‐activated protein kinase (MAPK) signalling pathways (Figure 7B, Supporting Information Table S6).

## 4 DISCUSSION

OA is a well‐known form of osteoarthrosis and is becoming an increasingly severe public health problem, whose underlying mechanism is not fully understood.^55^ Clarifying the molecular events during the pathogenesis of OA is an important endeavour. In recent years, lncRNAs have been reported to participate in the pathogenesis and progression of many diseases. Moreover, lncRNAs have been shown to be abnormally expressed in OA cartilage.^3^, ^12^ However, these previous studies mainly focused on a single genetic event or single cohort.^9^ Thus, in the present study, we integrated two data sets, GSE55235 and GSE55457, from the NCBI‐GEO database and utilized bioinformatics methods to analyse them thoroughly. We performed WGCNA to identify important modules and potential lncRNAs related to OA. WGCNA is a method widely used to cluster possible genes into different gene sets/modules.^27^, ^41^ The distinct advantage of WGCNA is that it focuses on the correlation between modules and clinical characteristics and has higher biological significance and reliability.^5^ In the current study, we found 11 separate modules and ultimately identified the green module as the most relevant module to OA clinical characteristics. In total, 49 significant differentially expressed genes with MM > 0.8 and GS > 0.2 were identified as hub genes in the green module. Among these hub genes, the top five genes and the only lncRNA were all downregulated in the synovial tissue of OA patients compared with their expression in normal samples in both the GSE55235 and GSE55457 data sets. These observations were also validated in an additional data set, GSE12021, which further confirmed the reliability of these markers.

NONHSAG034351 is the only lncRNA, attracted our attention. NONHSAG034351, also named LINC00312, is a long intergenic ncRNA (lincRNA) located on 3p25.3.^8^ LINC00312, as a novel tumour suppressor gene, has been associated with some cancers. Wang et al. found that it is downregulated in tissues of bladder cancer and can inhibit bladder cancer cell metastasis and invasion by regulating miR‐197‐3p.^47^ Qingqing Zhu et al. demonstrated that LINC00312 is also downregulated in paired tissues of nonsmall cell lung cancer, suppresses tumour proliferation, and promotes apoptosis by regulating HOXA5.^57^ Gang Li et al. reported that LINC00312 is downregulated in colorectal cancer tissues and inhibits colorectal cancer cell metastasis and proliferation by regulating miR‐21.^23^ LINC00312 also inhibits nasopharyngeal carcinoma cell invasiveness and proliferation.^48^ Here, our results showed that NONHSAG034351 is also downregulated in the synovial tissue of OA patients compared with normal samples based on three data sets, suggesting that it might play a role in OA. To our knowledge, the relevance of NONHSAG034351 has not been reported for OA. Thus, we further investigated its potential biological effects. The biological effects of lncRNAs are primarily exerted through the regulation of their coexpressed or target genes.^44^ We thus performed KEGG pathway and GO enrichment analysis of coexpressed genes in the green module to further investigate the biological functions of NONHSAG034351. GO analysis showed that biology processes such as the response to LPS, rhythmic process, response to molecules of bacterial origin, and angiogenesis were enriched in the green module. Among these, LPS has been reported to induce inflammation and has been used to induce arthritis in conjunction with collagen in experimental animal models.^28^, ^51^ Intriguingly, joint inflammation is one of the characteristics of clinical OA, which is considered to promote disease symptoms and aggravate disease progression in OA.^15^, ^34^ Studies have reported that LPS is a major risk factor for OA.^15^ Lai‐Bo Zhang et al. reported that calcitonin inhibits LPS‐induced apoptosis and inflammatory response through the MAPK/Wnt/NF‐κB pathways, which play a role in the treatment of OA.^54^ Oleocanthal was reported to suppress LPS‐mediated inflammatory responses and ADAMTS‐5, as well as MMP‐13 induction, via MAPKs/NF‐κB pathways in human primary OA chondrocytes.^38^ This is similar to our findings of KEGG analysis, which showed that the MAPK signalling pathway was enriched in the green module. Another study reported that the lncRNA THRIL downregulates microRNA‐125b in OA cells and promotes inflammatory injury induced by LPS.^25^ Thus, in our study, we concluded that NONHSAG034351 might take part in the biological process of LPS‐induced inflammation in OA; however, the related mechanism requires further investigation. In addition, we found that angiogenesis was enriched in the green module. Many previous studies have demonstrated the importance of angiogenesis in OA and that inhibition of angiogenesis could be a novel therapeutic approach to reduce pain and inflammation associated with OA.^17^, ^31^ For example, Leucine‐rich alpha‐2‐glycoprotein (LRG1) increases angiogenesis and recruits mesenchymal stem cells in the subchondral bone of OA joints to assist in angiogenesis‐coupled *de novo* bone formation, whereas the suppression of TNF‐α and LRG1 by lenalidomide could be an effective therapeutic approach for OA.^46^ Moreover, lncRNA maternally expressed 3 (MEG3) is decreased in OA and inhibits angiogenesis via p53 pathways.^22^ However, whether NONHSAG034351 participates in the process of angiogenesis in OA still needs to be investigated.

According to the KEGG pathway analysis, coexpressed genes in the green module mainly participate in the TNF, IL‐17, NOD‐like receptor, and MAPK signalling pathways. These pathways are mainly involved in inflammation and the progression of OA.^1^, ^4^, ^11^, ^13^ The MAPK signalling pathway, one of intracellular serine‐threonine protein kinase superfamily members, is the centre of multiple signal transduction pathways.^18^ It has been suggested that the MAPK pathway activation results in the overexpression of proinflammatory cytokines, chemokines, and signalling enzymes in human OA chondrocytes, which play a significant role in the progression of OA.^4^ Blockage or inhibition of the activation of MAPK signalling can inhibit inflammatory cytokine production in OA chondrocytes to prevent the degradation of bone and cartilage.^39^ The TNF signalling pathway is another important pathway in the regulation of inflammation. Studies have shown that miR‐17 overexpression suppresses TNF receptor‐associated factor 2 (TRAF2) expression and is correlated with cellular inhibitor of apoptosis 2 (cIAP2), thus suppressing TNF‐α signalling pathways and downstream inflammatory proteins.^1^ In classical TNF‐α signalling, TNF receptor 2 (TNF‐R2) activates the NF‐κB and MAPK pathways.^29^ In addition, the MAPK and TNF‐α signalling pathways have also been reported to participate in angiogenesis.^11^, ^53^ For example, nerve growth factor promotes the expression of FGF2 in vitro in human chondrocytes, increasing angiogenesis via the phosphoinositide 3‐kinases (PI3K) or ERK/MAPK signalling pathways, which could be the reason for increased vascular growth from the subchondral bone in OA. Thus, we concluded that, in our study, NONHSAG034351 might regulate the biological process of OA through an intricate regulatory network comprising the MAPK and TNF‐α signalling pathways.

In summary, using profile data sets from multiple cohorts and WGCNA, we have identified gene modules associated with OA clinical traits for the first time. NONHSAG034351, the only hub‐lncRNA in the OA‐related green module, was downregulated in the synovial tissue of OA patients and might play an important role in the pathological progression of OA. Our findings suggest novel targets for further investigation related to both the molecular mechanisms of OA and potential therapeutic interventions, which has obvious important clinical implications.

## AUTHOR CONTRIBUTIONS

Xiaolong Shui and Yi Wang wrote the manuscript. Xiaolong Shui and Qipeng Xie analysed the raw data. Chengwei Zhou and Jianzhong Kong collected the raw data. Yi Wang designed the entire study.

## CONFLICT OF INTEREST

The authors declare that they have no conflict of interest.

## Data Availability

The raw data sets (Data sets GSE55235, GSE55457, and GSE12021) used in this study were downloaded from the GEO database (http://www.ncbi.nlm.nih.gov/geo/).

## DATA AVAILABILITY STATEMENT

The raw datasets (Datasets GSE55235, GSE55457, and GSE12021) used in this study were downloaded from the GEO database (http://www.ncbi.nlm.nih.gov/geo/).
